# Fungal diversity in the soil Mycobiome: Implications for ONE health

**DOI:** 10.1016/j.onehlt.2024.100720

**Published:** 2024-04-16

**Authors:** Andreas Yiallouris, Zoi D. Pana, Giorgos Marangos, Ioanna Tzyrka, Spyridon Karanasios, Iliana Georgiou, Kyriaki Kontopyrgia, Eleni Triantafyllou, Danila Seidel, Oliver A. Cornely, Elizabeth O. Johnson, Stavros Panagiotou, Charalampos Filippou

**Affiliations:** a School of Medicine, European University, Cyprus; b Medical innovation center (MEDIC), School of Medicine, European University, Cyprus; c Division of Medical Education, School of Medical Sciences, Faculty of Biology, Medicine and Health, University of Manchester; d University of Cologne, Faculty of Medicine and University Hospital Cologne, Translational Research, Cologne Excellence Cluster on Cellular Stress Responses in Aging-Associated Diseases (CECAD), Cologne, Germany; e University of Cologne, Faculty of Medicine and University Hospital Cologne, Department I of Internal Medicine, Center for Integrated Oncology Aachen Bonn Cologne Duesseldorf (CIO ABCD) and Excellence Center for Medical Mycology (ECMM), Cologne, Germany; f German Centre for Infection Research (DZIF), Partner Site Bonn-Cologne, Cologne, Germany

**Keywords:** Soil mycobiome, One health, Climate change, Antifungal resistance, Ecosystem functioning, Soil biodiversity

## Abstract

Today, over 300 million individuals worldwide are afflicted by severe fungal infections, many of whom will perish. Fungi, as a result of their plastic genomes have the ability to adapt to new environments and extreme conditions as a consequence of globalization, including urbanization, agricultural intensification, and, notably, climate change. Soils and the impact of these anthropogenic environmental factors can be the source of pathogenic and non-pathogenic fungi and subsequent fungal threats to public health. This underscores the growing understanding that not only is fungal diversity in the soil mycobiome a critical component of a functioning ecosystem, but also that soil microbial communities can significantly contribute to plant, animal, and human health, as underscored by the One Health concept. Collectively, this stresses the importance of investigating the soil microbiome in order to gain a deeper understanding of soil fungal ecology and its interplay with the rhizosphere microbiome, which carries significant implications for human health, animal health and environmental health.

## 1 Introduction

Fungi comprise a vast and intricate eukaryotic domain, encompassing between 2.2 and 3.8 million species that predominantly inhabit terrestrial ecosystems [1]. This remarkable diversity stems from their exceptionally plastic genomes and, consequently, their notable ability to occupy new environments, leverage unique resources, and forge innovative associations [2]. Anthropogenic impacts from globalization, such as soil acidity and heavy metal contamination resulting from industrial activities, have compromised the soil ecosystem's integrity and function. This in turn has affected fungal biodiversity. Fungi have developed potent adaptations to survive in these often highly toxic and extreme physical conditions, such as high salt concentrations, extreme pH levels, and the presence of heavy metals. For instance, the basidiomycetous fungus *Cryptococcus neoformans* thrives in the otherwise lethal radioactive environment near the Chernobyl reactor, attributed to its robust DNA repair mechanisms and effective antioxidant defences [3].

The intricate interactions between animals, humans, and ecosystems, intensified by rapid urbanization, growing human and livestock populations, and evolving farming systems, have escalated disease spillover. Habitat destruction, the globalization of animal product trade, antimicrobial resistance (AMR), climate change, and the misuse of natural resources have led to ecosystem degradation, weakening natural defences against disease emergence [4]. Habitat fragmentation, caused by urbanization and exploitation, has decreased biodiversity, promoted disease vectors, and exacerbated disease spillover across wildlife, domestic animals, and humans [5]. The latter emphasizes the interconnectedness of human, animal, and environmental health, encapsulated in the One Health concept.

Considering that over 300 million individuals worldwide are afflicted by severe fungal infections and nearly 1.5 million people die from these diseases annually [6], there is an increasing need to be aware of the importance of addressing emerging health threats, antifungal resistance, and fungal infection management. This scoping review fundamentally posits that the diversity of fungi in the soil mycobiome is crucial for a functioning ecosystem and, by extension, public health. It aims to map the current literature on the contributions of soil microbial communities to plant, animal, and human health, emphasizing the One Health concept. We assess how soils, affected by anthropogenic environmental factors, can become sources of both pathogenic and non-pathogenic fungi, posing subsequent threats to public health. The evidence presented here underscores the importance of studying the soil microbiome to gain a deeper insight into soil fungal ecology and its relationship with the rhizosphere microbiome. This carries significant implications for human, animal, and environmental health.

### 1.1 Soil mycobiome in plant health and sustainability

Microbial communities form diverse and intricate microbiomes, with soil microbes such as arbuscular mycorrhizal fungi (AMF) and nitrogen-fixing bacteria (NFB) contributing to the acquisition of limiting nutrients like nitrogen (N) and phosphorus (P) for plants [7]. A balance of NFB, AMF, plant growth-promoting rhizomicrobes (PGPR), biocontrol microbes, and Protozoa enhances plant growth and health. The development of arbuscules in the host plant's root cortex provides a large surface area for accessing otherwise unavailable nutrients [8]. In agri- and horticultural ecosystems, soil fungi play pivotal roles, broadly categorized into three functional groups based on their ecological roles and life modes: 1) symbiotic/mycorrhizal, including AMF (e.g *Gigaspora margarita*, *Scutellospora calospora, Acaulospora* sp.) which enhance plant growth, nutrient cycling, and stress tolerance. Symbiotic fungi like *Glomus* sp. and *Trichoderma* sp. play a biocontrol role by suppressing fungal pathogens; 2) soil-borne root pathogens, comprising genera like *Fusarium*, *Verticillium*, and *Rhizoctonia*, which are significant plant health threats globally; and 3) saprotrophic fungi, the largest group, crucial for organic matter decomposition and carbon recycling. They employ a wide array of metabolic processes and extracellular enzymes, vital for breaking down complex biological entities like lignocellulose and chitin, exemplified by species such as *Piptporus betulinus* and *Fomitopsis pinicola* [9].

The diversity of fungi in soil is intrinsically linked to the floral composition and organic matter in the soil (Supplementary Table 1). Imbalances in the plant microbiome can lead to certain plant diseases [10]. For example, *Fusarium* wilt in tomatoes is associated with decreased microbial diversity and altered fungal communities in the tomato rhizosphere [11]. Factors like unpredictable weather, drought, poor soil fertility, pests and diseases can disrupt the soil ecosystem. Pathogens such as *Gaeumannomyces graminis*, *Fusarium pseudograminearum*, and *Rhizoctonia solani* [10,12] have contributed to a 50% annual decline in wheat production. In contrast, microbial diversity and complexity are necessary for the balanced functioning of the soil ecosystem. The presence of beneficial microbes may provide resilience against disruptions [13].

Endophytic fungi (EF) protect the host plant from other fungal pathogens and/or insect herbivores by producing toxic compounds or by modifying the host plant's defence response to enhance pest and pathogen resistance. For example, EF from the genus *Epichloë* are widely exploited as protective agents for pasture pest management [14]. *Beauveria*, recognized as the anamorphic state of the genus *Cordyceps*, plays a protective role in plant health, notably against plant-pathogenic species such as *Fusarium* spp., *Rhizoctonia solani*, and *Pythium myriotylum*. [15,16]*.*

Although highly diverse microbiomes on small spatial scales may seem functionally redundant, functional redundancy is an important feature of biodiversity. Communities with higher microbial richness perform better because they can ensure the maintenance of functioning under varying environmental conditions. Data supports the idea that a taxonomically rich soil microbiome underpins soil multifunctionality by ensuring greater association complexity. Microbial interkingdom associations are vital for driving ecosystem functioning, and these unseen synergisms might be more widespread and ecologically significant for the soil microbiome's functioning than previously thought [17].

#### 1.1.1 Fungi impact on plant health

Soil fungi play a vital role in promoting plant health and suppressing diseases. Mycorrhizal fungi (MF) extend the reach of plant roots, accessing nutrients from a larger soil volume, thereby improving plant growth and yield [18]. Soil fungi decompose organic matter, releasing essential nutrients that become available for plant uptake. This process, known as decomposition or mineralization, contributes to the overall fertility of agricultural soils. Fungal-driven decomposition plays a crucial role in breaking down complex organic compounds and converting them into forms that can be readily utilized by plants [19].

Antagonistic fungi protect plants against pathogenic organisms through various mechanisms such as competition for resources, production of antifungal compounds, and inducing plant defence responses [20]. For instance, *Trichoderma* species are well-known biocontrol agents that can protect plants against a range of fungal pathogens. These fungi colonize the root zone, establishing a protective barrier and inhibiting the growth of harmful pathogens. Moreover, some soil fungi have been found to induce systemic resistance in plants, enhancing their ability to withstand diseases [21].

The impact of soil fungi extends beyond individual plants and significantly affects sustainable agriculture. One compelling example of soil fungi's pivotal role in agriculture is the ongoing Banana Pandemic (also known as Panama disease or Fusarium wilt). This grave crisis disrupted global agriculture and raised serious concerns about food security. The Banana Pandemic is caused by the fungus *Fusarium oxysporum* f. sp. *cubense* (FOC). This pathogen infiltrates the vascular system of banana plants, blocking water and nutrient flow, leading to wilting and eventual death. The disease proliferates through contaminated soil, water, and infected plant material, which makes containment challenging [22].

The consequences of the Banana Pandemic are far-reaching, with significant economic and environmental implications [23]. The environmental impact of the Banana Pandemic resulting from the extensive use of fungicides, which not only harm ecosystems, but also contribute to pesticide resistance, was also significant. This was compounded by loss of banana plantations leading to reduced biodiversity and threatening the habitat of various organisms that depend on banana crops for survival [24].

#### 1.1.2 Factors impacting soil microbiome

Using both organic fertilizers (OF) and chemical fertilizers (CF) produces higher crop yields than using CF alone, [25] enhances soil enzymatic activity [[26], [27], [28]], and improves soil quality. The combined application of OF and CF can enhance soil microbial activity, interactions, biomass [29] and increase fungal diversity, particularly beneficial fungi such as *Aureobasidium pullulans* and *Candida pyralidae*. These fungal species can act as biocontrols against fruit diseases and spoilage [29]. In contrast, soils treated solely with CF show an increased abundance of pathogenic fungi such as *Aspergillus*, *Botrytis*, and *Cryptococcus [**30**]*, and pathogenic fungal taxa such as Chaetomiaceae, Chaetothyriales, *Cyphellophora*, and *Bipolaris*. These are associated with diseases like grape black rot, grey mold, and specific human infections [31].

Conventional tillage (CT), which involves techniques like chisel ploughing and disc ploughing, is known to negatively impact the structure and function of soil microbial communities, including bacterial and fungal diversity [32,33]. CT decreases the soil's binding capacity, heightening susceptibility to pollutant erosion, such as chemical pesticides through surface runoff [34]. Consequently, soil enzyme activity is altered. With CT, the hyphal network of mycorrhizae can be disrupted, reducing the abundance of mycorrhizae and their associated beneficial effects on nutrient acquisition and soil organic carbon protection [34]. Extensive CT can increase the prevalence of saprophytic fungi [35] and plant pathogenic fungi, like *Aspergillus niger* and *Aspergillus terreus*, and also foster the plant pathogen *Fusarium* spp. [33].

In contrast, conservation agriculture focuses on reducing soil disruption while promoting crop productivity through methods like crop rotation, cover crops, and reduced or no-tillage (NT). NT enhances soil physical properties, leading to decreased soil compaction, increased thermal conductivity [36] and elevated concentration of soil organic matter [33]. NT has been linked with a 14% rise in the activity rates of B-glucosidase and b-glucosaminidase enzymes, both soil health indicators. It also boosts soil carbon, nitrogen, and phosphate availability compared to CT. Reduced tillage is correlated with an increase in microbial biomass carbon and higher bacterial and fungal abundances compared to CT [35]^,^ [37,38]. Notably, the abundance of MF, actinomycetes, and Gram-positive bacteria significantly rises with NT, facilitating nutrient translocation [39,40]. Additionally, NT promotes beneficial nematophagous fungi like *Myrothecium verrucaria* and *Paecilomyces*, providing protection to the host plant.

Urbanization significantly influences the soil microbiome composition. While soil archaea and bacteria in urban locations maintain their biodiversity, soil fungal richness substantially diminishes in these areas [[41], [42], [43]]. Increased urbanization results in a drop in macromycete species and functional richness [41,42]. Fungal communities converge in ruderal sites, places in cities where soil has been disrupted due to construction or dismantling activities. Analysis shows this convergence is more attributed to human-induced soil disturbance than city-related environmental factors, such as rising temperatures. A decrease in the abundance and richness of ectomycorrhizal fungi correlates with this convergence. This observation is attributed to the removal of woody perennials associated with this fungal symbiont in the Northern Hemisphere [44]. Increased urbanization pressure induces convergence of the soil microbial community (SMC) in urban forests, whereas, rural forests show SMC dissimilarity [45]. The authors concluded that a significant overlap of shared taxa, rather than biodiversity loss, drove the SMC convergence in urban forests, as species richness and SMC abundance did not vary among the forest types [45].

### 1.2 Climate change and fungal threats

Pathogenic fungi represent a diverse group of organisms that are widely dispersed in the environment, thriving in various substrates including soil, which is a primary source for many fungi that cause diseases, or mycoses. Commonly found in both soil and air, species like *Aspergillus* spp., *Lichtheimia corymbifera*, *Lichtheimia ramosa*, and *Rhizopus arrhizus*, pose persistent challenges due to their ability to affect a broad range of hosts [46,47]. With the progression of climate change, there is a notable impact on the development, proliferation, and distribution of these fungal pathogens, leading to alterations in their pathogenicity and patterns of antifungal resistance. The changing climate imposes new environmental pressures, facilitating the emergence of novel pathogens which can invade new geographic areas, infect previously unaffected hosts, and show increased incidence of diseases. These emergent pathogens consequently pose risks to agricultural yields, public health, and the biodiversity of wildlife [48].

Climate change is anticipated to increase the Earth's temperature by several degrees in the foreseeable future, narrowing the gap between environmental and mammalian base temperatures. This could potentially lead to the development and spread of various fungal diseases [48,49]. New, highly virulent fungal strains are believed to have evolved due to climate change. For instance, *Puccinia striiformis*, the rust fungus responsible for one of the most destructive wheat diseases worldwide, previously preferred colder regions but is now invading warmer areas [50,51]. Since 2000, more aggressive, and heat-tolerant strains like Pst1, Pst2, and “Warrier” have emerged, replaced former strains and expanded into new territories [48,51]. Another significant concern is Fusarium head blight (FHB), a disease severely affecting wheat and other cereal crops. Infections from the *Fusarium graminearum* species complex (FGSC) lead to reduced cereal yield and quality, threatening food security [52]. Outbreaks typically occur during warm, humid weather, causing yield losses of up to 75% [53]. In the past two decades, some temperate regions have seen a shift from *Fusarium culmorum*, which prefers cooler, wetter conditions, to *F. graminearum*, which thrives in warmer, humid conditions. *F. graminearum* produces more mycotoxins, and their production seems to increase with higher temperatures and water stress, potentially affecting human and animal health [48,54]. As the climate continues to change, these evolving fungal pathogens are not just an agricultural concern; they increasingly pose direct threats to human health, especially in the aftermath of natural disasters that provide ideal conditions for their spread and infection.

#### 1.2.1 Impact on human health

Climate change impacts weather patterns, leading to floods, droughts, intensified tropical storms, and tornadoes, all of which can significantly affect human health. A growing understanding exists regarding the association between natural disasters and ensuing fungal infections [48]. Natural disasters can displace and spread disaster-related fungi, leading to lung and soft tissue infections from fungal species that are usually rare, as seen in the coccidioidomycosis outbreak following the 1994 Northridge earthquake in California, USA [55]. This outbreak is one of the few recorded instances of an infectious disease outbreak directly tied to a geophysical disaster. Coccidioides spores were believed to have been aerosolized due to the earthquake and the subsequent widespread dust clouds. In Ventura County, California, 203 coccidioidomycosis cases associated with the outbreak were reported, with dust exposure being a major factor linked to acute illness (Supplementary Fig. 1). However, fungal infection may not have been the initial diagnosis; 93% of patients were treated with at least one antibacterial drug before coccidioidomycosis was identified [55].

Additionally, damage to local healthcare infrastructure can hinder the proper cleaning of contaminated wounds with sterile solutions or the immediate treatment of injuries with topical or systemic antibiotics. Mucormycosis, caused by Mucorales fungi, is perhaps the most well-known example of post-disaster fungal tissue infection, leading to necrotizing fasciitis, with roughly 30% case-fatality rates [56]. The first recorded case of disaster-related mucormycosis occurred after the volcanic eruption in Armero, Colombia, in 1985, which led to an estimated 23,000 fatalities and around 4500 injuries. A study of 38 patients with necrotizing wounds hospitalized post-disaster revealed that eight had infections caused by the mucormycete *Rhizopus arrhizus* [48,57]. Following the 2004 Indian Ocean tsunami, an outbreak of *Aspergillus*-associated meningitis was observed, linked to spinal anesthesia used for cesarean section deliveries in Sri Lanka. Initial treatments targeted bacterial meningitis, but post-mortem examinations revealed *Aspergillus*, leading to treatments with amphotericin B and voriconazole for respective other patients. The investigation found that syringes were contaminated with *A. fumigatus* likely because of poor storage conditions in a humid warehouse [58]. The likelihood of wound infections after a natural disaster is significant when wounds come into contact with muddy water, soil, or debris [59].

The rhizosphere is a dynamic ‘microbial hot-spot,’ characterized not only by its high nutrient content and enhanced bacterial abundances but also by a diverse array of fungi playing crucial roles. Various bacterial genera, such as *Burkholderia*, *Enterobacter*, *Herbaspirillum*, *Ochrobactrum*, *Pseudomonas*, *Ralstonia*, *Staphylococcus*, and *Stenotrophomonas*, harbor root-associated strains capable of interacting with plant and human hosts. Similarly, the rhizosphere is rich in fungal species like *Trichoderma*, *Rhizoctonia*, and *Mycorrhiza* participating in symbiotic relationships with plants but also have been found to have direct or indirect effects on human health, either through their role in food production or potential pathogenicity [60].

The processes underlying rhizosphere colonization and antagonistic behavior against plant pathogens closely resemble those responsible for human organ and tissue colonization and pathogenicity, with some species displaying multiple antibiotic and antifungal resistances [61].

*Candida auris*, first identified in 2009 from an ear infection in Japan, is believed to have an environmental origin, possibly birds and climate change, although it may be related to increased human contact due to expanded farming and aquaculture, and fungicide contamination. The complications brought on by the multidrug-resistant microorganism *C. auris* underscore several concerns: *C. auris* not only leads to heightened illness and fatality rates among impacted individuals, but it is also tough to eliminate from healthcare facilities, despite the implementation of rigorous infection-control measures [48,62,63].

#### 1.2.2 Impact on animal health

Fungi extensively influence the animal kingdom, infesting or infecting nearly all animal species, with insects being particularly susceptible. Their role in population control of insects is well-documented. Yet, it's interesting to note the relative rarity of fungal infections in vertebrates, with only about 625 fungal species identified as vertebrate pathogens. This is likely due to the sophisticated innate and adaptive immune responses and higher body temperatures of endothermic vertebrates, which resist fungal pathogens. Rabbits, for example, show remarkable resistance to systemic infections by *C. neoformans* unless their immune system is compromised. Despite this, the increasing prevalence of fungal diseases in animals is a global concern, often linked to weakened immunity [49].

Fungal infections in animals can also arise from mycotoxin contamination in feed, with secondary fungal metabolites inducing both immediate and chronic health problems, including stunted growth in juveniles. The situation could worsen with climate change, which may enhance conditions for mycotoxin-producing fungi [64]. Among the significant fungal threats to animals, especially birds, is aspergillosis. It is the main cause of morbidity and mortality in wild birds and poultry, causing both ecological and economic repercussions. Birds play a significant role in spreading *Aspergillus* spp., including azole-resistant strains, due to their frequent movement across different habitats. Aspergillosis affects not only birds but also invertebrates such as corals, honeybees, and reptiles. In mammals like horses and cows, *A. fumigatus* leads to various conditions, including mycosis, pneumonia, and gastroenteritis. Immunocompromised cats and dogs are also vulnerable to aspergillosis, which can result in a range of infections [64].

Other opportunistic fungal pathogens, like Mucorales, are known to infect cattle, horses, birds, cats, and dogs, leading to conditions such as ruminitis and lymphadenitis in cattle, respiratory and gastrointestinal issues in horses and birds, and enteritis or systemic mucormycosis in cats and dogs [64]. Wild species are not exempt, with reports of infections in dolphins, bison, and seals.

Additionally, amphibians face a critical threat from the pathogenic fungus *Batrachochytrium dendrobatidis* (Bd), which has caused dramatic population declines worldwide. Climate change is expected to exacerbate this issue by expanding the pathogen's habitat and increasing amphibian vulnerability [48]. Likewise, *Pseudogymnoascus destructans* a psychrophilic fungus, has ravaged North American bat populations through white-nose syndrome by exploiting on the bats' lower body temperatures during hibernation [65]. *Cryptococcus deuterogattii*, once a member of the *C. gattii* complex, previously associated with warmer climates, has now adapted to temperate zones, causing infections in both animals and humans in North America, with its spread linked to human activities and possibly climate change [48] (Table 1).

**Table 1:** List of soil fungi reported as human or animal pathogens.

Fungi	Climate Zone	Ecological Role	Local Environmental, Conditions	Pathogenicity to humans/ animals	Transmission	Citation
*Acremonium* spp.	Various	Opportunistic pathogen/ Symbiont	Moderate organic matter content	Eumycetoma, onychomycosis, and hyalohyphomycosis	Inhalation or cutaneous inoculation	[86]
*Alternaria* spp.	Various	Decomposer	High moisture and low temperature	Subcutaneous phaeohyphomycosis, mycotic keratitis and allergic reactions	Inhalation	[86]
*Apophysomyces variabilis*	Tropical and sub-tropical	Decomposer	Thermotolerant species / human and bat pathogen	Mucormycosis	Inhalation	[96]
*Aspergillus* spp.	Tropical	Decomposer	High organic matter content	Aspergillosis, Aspergillomas	Inhalation	[85]
*Aureobasidium* spp.	Various	Endophyte	High moisture	Hypersensitivity pneumonitis, catheter-related fungemia and onychomycosis	Inhalation or cutaneous inoculation	[91]
*Batrachochytrium dendrobatidis*	Various	Amphibian pathogen	Aquatic fungus	Infects the keratinized skin of amphibians	Direct contact	[96]
*Candida* spp.	Various	Opportunistic pathogen	Associated with human activity	Candidiasis, Esophageal candidiasis, Hepatosplenic candidiasis	Direct contact	[91]
*Chaetomium* spp.	Moderate temperature	Decomposer	Alkaline environment	Allergic reactions, respiratory infection and onychomycosis	Inhalation or cutaneous inoculation	[91]
*Cladophialophora bantiana*	Tropical and sub-tropical	Opportunistic pathogen	Acidic, glucose-rich environment	Cerebral phaeohyphomycosis in cats, dogs and humans	Inhalation or post-inoculation by thorns	[91]
*Cladosporium* spp.	Temperate	Airborne, decomposer	High humidity, shaded areas	CNS infections, Cutaneous infections	Inhalation	[91]
*Cryptococcus deuterogattii*	Tropical and sub-tropical	–	Soil, decaying wood, tree hollows, bird droppings	Pulmonary cryptococcosis (lung infection), basal meningitis, and cerebral cryptococcomas	Inhalation	[48,96]
*C. neoformans*	Various	–	Soil, decaying wood, tree hollows, bird droppings	Pulmonary cryptococcosis (lung infection), cryptococcal meningitis	Inhalation	[96]
*Emmonsia parva*	Various	Decomposer	Soil saprotroph	Adiaspiromycosis in small mammals	Inhalation	[96]
*Epidermophyton floccosum*	Various	Anthropophilic dermatophyte	Saprotroph	Tinea pedis, tinea cruris, tinea corporis and onychomycosis.	Direct or indirect contact	[96]
*Exserohilum rostratum*	Tropical, Subtropical	Decomposer	High organic matter and moisture	Fungal Meningitis, Fungal Sinusitis	Inhalation	[88]
*Fonsecaea monophora*	Tropical and sub-tropical	Decomposer	Soil saprotroph	Chromoblastomycosis	Cutaneous inoculation	[96]
*Fusarium* spp.	Subtropical	Plant pathogen	Irrigated, crop rotation	Fusariosis	Inhalation, Skin break	[92]
*Histoplasma capsulatum*	Various	Decomposer	Contaminated soils with birds and bats droppings, nitrogen & phosphorous rich soil	Histoplasmosis	Inhalation	[87]
*Lomentospora prolificans*	Various	Emerging opportunistic pathogen	Soil, decaying organic matter and contaminated water	Localized to disseminated infections in humans and animals	Skin break	[96]
*Madurella grisea*	Subtropical	Human pathogen	Soil	Eumycetoma	Skin break	[96]
*Malassezia furfur*	Various	Component of skin microbiome	Commensal organism	Seborrhoeic dermatitis, dandruff, pityriasis versicolor, and tinea circinata,	–	[96]
*Mucor* spp.	Temperate	Saprotrophs, Nutrient Cycling	High organic matter, High humidity	Mucormycosis	Inhalation, Skin break	[98]
*Pithomyces chartarum*	Tropical and Subtropical	Decaying matter	Soil saprotroph	Facial eczema in sheep and cattle	Direct contact with dead plant material	[96]
*Pseudogymnoascus destructans*	Low temperature	Soil, decaying matter, caves and mines	Psychrophilic / opportunistic pathogen on bats	White-nose syndrome (WNS)	Inhalation	[97]
*Purpureocillium lilacinum*	Various	Nematode pathogenic fungus	High organic matter, High humidity, Nematode populations	Pulmonary, soft-tissue and disseminated infection in immunocompromised patients	Inhalation, Direct contact	[96]
*Rhizopus* spp.	Humid	Mutualist	High moisture content	Mucormycosis	Inhalation, Skin break	[89]
*Sporothrix* spp.	Various	Wood decomposer / opportunistic pathogen	Soil saprotroph	Sporotrichosis “rose gardener's disease” in domestic animals and humans	Inhalation or skin break puncture	[68]
*Talaromyces marneffei*	Tropical	Opportunistic pathogen	High moisture	Talaromycosis	Inhalation	[91]
*Thyridium* spp.	Various	Plant and human pathogen	Saprobic	Superficial and systemic infections	Inhalation	[93]
*Trichoderma* spp.	Subtropical	Biocontrol agent	High nutrient content	Invasive pulmonary infection, CNS infection	Inhalation	[90]
*Trichophyton* spp.	Various	Anthropophilic dermatophyte	Saprotroph	Tinea corporis, tinea capitis, tinea pedis and tinea unguium	Direct or indirect contact	[96]
*Trichosporon* spp.	Tropical	Decomposer / opportunistic pathogen	High organic matter and moisture	Superficial and systemic (hypersensitivity pneumonia)	Close contact	[95]
*Veronaea botryosa*	Various	Wood decomposer	Saprobic	Phaeohyphomycosis in captive White's tree frogs (*Litoria caerulea*)	Land-based (contaminated water or vegetable)	[94]

The complexity of the impact of fungi on animal health encompasses ecological, immunological, and environmental aspects, including the influence of climate change. The dissemination of certain diseases is propelled by both natural processes and human activities, underscoring the need for vigilant surveillance and proactive strategies to mitigate their effects on wildlife, livestock, and by extension, human populations.

### 1.3 One health concept

Considering plant health is a critical component of environmental health, and thus an integral part of the One Health concept, human pathogenic fungi and yeasts hold significant relevance. This is especially evident in crop protection. Traditional agricultural fungicides often contribute to antimicrobial resistance (AMR), one of today's most pressing challenges, underscoring the fundamental premise of One Health. A case in point is the observed link between the use of agricultural fungicides, which are structurally similar to medical triazoles and azole resistance in *A. fumigatus,* the main cause of invasive pulmonary aspergillosis.

**Fig. 1:**
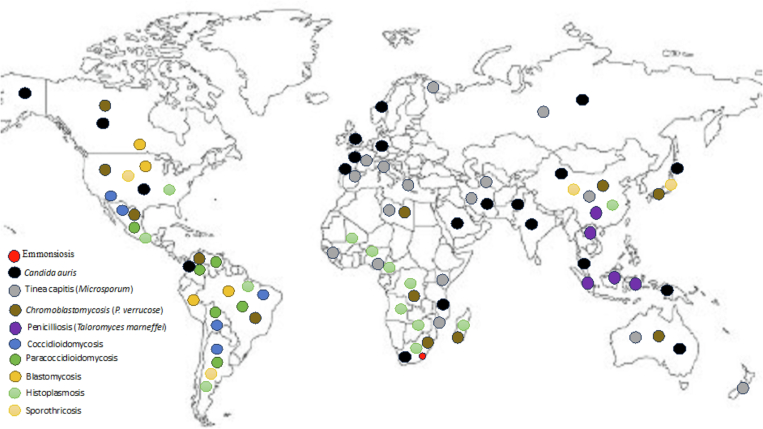
Fungal diseases & geographical location. *Sporotrichum*, *Phialophora*, *Microsporum*, *Candida*, *Histoplasma*, *Coccidioides*, *Paracoccidioides*, *Talaromyces*, and *Blastomyces* species are pathogenic fungi with saprophytic life cycles, living and thriving in various natural habitats. *Sporotrichum schenckii*, the agent of sporotrichosis or “Rose Gardener's Disease” is prevalent in soils with decaying vegetation and is also found in many warm-blooded animals, posing zoonotic transmission risks. Depending on the strain, it can cause either lymphatic sporotrichosis or localized skin lesions. *Phialophora verrucosa*, causes chromoblastomycosis, a stubborn skin infection found in tropical regions. This fungus has been isolated from old boards and soil. Similarly, *Microsporum* spp., which causes Tinea capitis, as well as *Trichophyton* spp., and *Candida* spp., which are both found in soil and decaying vegetation and can cause various mucosal infections. Several dimorphic fungi with saprophytic life cycles are geographically confined to the Americas. Examples include *Histoplasma*, *Coccidioides*, *Paracoccidioides*, *Talaromyces*, and *Blastomyces* species. While they often cause mild pneumonia, they can cause severe infections in immunocompromised individuals. Specifically, *C. immitis* and *C. posadasii* are desert-dwelling fungi found primarily in the US states of Arizona and California. *Histoplasma capsulatum* is associated with bird and chicken excrement in humid, tropical zones. *Paracoccidioides brasiliensis* thrives in acidic soils in South and Central America, especially where coffee and sugar canes are cultivated. *Blastomyces dermatitidis* is predominant in the wet soils of the Eastern US. *T. marneffei*, a fungus endemic to Southeast Asia, is closely associated with bamboo rats and the soil of their burrows. Lastly, disseminated emmonsiosis linked to HIV and caused by the new *Emmonsia* species is known in South Africa.

Fungi have also caused unexpected, large-scale fatalities in animal populations. Diseases such as white-nose syndrome in bats and chytridiomycosis in amphibians are prominent examples [66]. Cross-resistance in pathogenic yeasts like *Candida* spp. and *Cryptococcus* spp. has been attributed to fungicide treatments on various crops. Moreover, certain insecticides have been reported to induce azole resistance in pathogenic *Candida spp [**67**]*. New-age One Health-associated fungal risks include the recent outbreak of *Sporothrix brasiliensis* in humans in South America, which has been linked to close contact with cats, and the increasing instances of fungal keratitis resulting from agricultural activities conducted without proper eye protection. There is also the consistent occurrence of sporotrichosis linked to unprotected horticultural activities and the emergence of diseases like histoplasmosis and chromoblastomycosis in newly identified geographic regions [68] (Fig. 1).

The misuse of antibiotics is correlated with the rise in antibiotic resistance (ABR). This resistance pervades human communities, livestock, and their environments, leading to the establishment of resistance reservoirs persistent in the environment [69]. ABR spreads through multiple environmental reservoirs such as water, soil, hospitals, industrial and agricultural waste, and other contaminated biological niches, allowing pathogens carrying resistance genes to be transported within or among individuals, livestock, and the surrounding environment [70]. Antibiotic-resistant bacteria can transmit their resistance genes to normal human gut bacteria and subsequently to pathogens causing human diseases. This presents a significant risk related to antibiotic usage in animal husbandry, termed the ‘reservoir hypothesis’ [71]. Freshwater systems are also susceptible to antibiotic pollution from sources, including fertilizer runoff, wastewater discharge, and seepage from nearby farms. In these environments, the presence of antibiotics and a high concentration of active bacteria promote the emergence of antibiotic resistance genes through horizontal gene transfer (HGT), leading to the evolution of resistance [70].

#### 1.3.1 Emerging fungal diseases

In October 2022, the WHO established the Fungal Priority Pathogens List (WHO FPPL) to identify and prioritize the most critical fungal pathogens based on their threat to public health. Only one national infectious disease threat priority list that included fungal pathogens was discovered, specifically the US CDC Priority Threat List (2019), which emphasized three fungal “groups”: *C. auris*, antifungal-resistant Candida, and azole-resistant *A. fumigatus*. Furthermore, India prioritized mucormycosis under the notifiable disease category in 2021 due to the largest outbreak to date, which was associated with the COVID-19 pandemic [72]. The list serves as a vital resource for directing research, surveillance, and policy decisions concerning fungal infections. By employing the multicriteria decision analysis (MCDA) approach, the WHO ensures a thorough and flexible evaluation framework that considers the evolving landscape of fungal threats. Regular updates to the list and increased collaboration among global health organizations will be essential for effectively addressing emerging and re-emerging fungal infections worldwide [73].

**Fig. 2:**
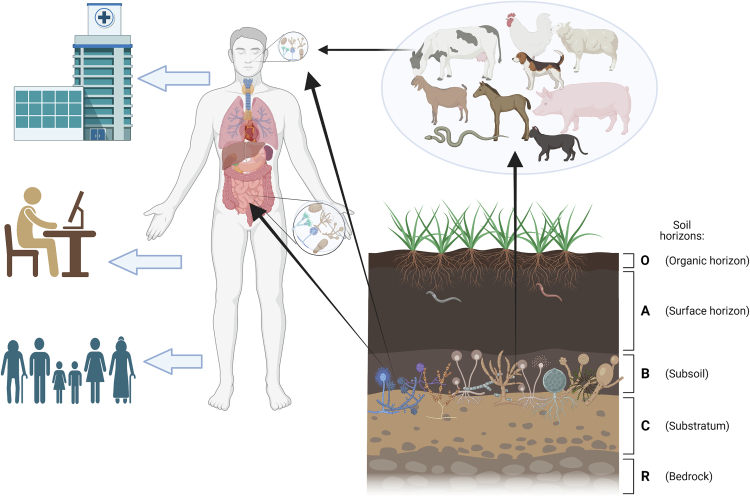
Soil fungi migration to healthcare facilities, residential homes, and workplaces. The transition dynamics of soil fungi to anthropogenic environments, specifically healthcare facilities, residential areas, and workplaces, with an emphasis on implications for the One Health approach. The migration of fungi from their natural soil habitats into these environments can be facilitated through activities led by humans or by animal-assisted dispersal. Numerous factors, including soil disturbances resulting from construction or farming activities, erosion due to wind and water, and certain animal behaviours such as burrowing and foraging, can all promote the release and spread of fungal spores. These spores may become airborne or transported via carriers. As the site of the most diverse and intricate microbiome on Earth, soil serves as a critical microbial reservoir. Bulk soil is likely the primary source of plant endophytic microbiota, contributing over two-thirds of the overall bacterial and fungal diversity. Dietary choices play a significant role in determining the oral and gut microbiome composition of both humans and animals, with plant microbiota, including microorganisms sourced from soil, making their way into human and animal gut microbiota. Frequent exposure to soil occurs in farmers or farm animals, and soil particles containing microorganisms can also be inhaled through dust. The act of deliberately consuming soil or clay, known as geophagy, is observed in animals such as sheep, gorillas, bats, and parrots, as well as in humans. The figure emphasizes the diversity of fungi that can infiltrate these settings, including *Aspergillus, Cryptococcus*, *Candida*, *Fusarium*, *Pneumocystis*, and *Coccidioides* species, which have been associated with significant health issues in immunocompromised individuals (bottom right). Once introduced into indoor settings, these fungi may find conducive conditions to grow and spread. Prolonged exposure to these fungi may have both direct health implications such as potential allergic reactions or fungal infections (cutaneous or invasive), and indirect effects, which could exacerbate existing health conditions or contribute to poor indoor air quality (arrows leaving from the main human figure connecting workplace, healthcare and residential homes). The essential need for a cooperative, interdisciplinary approach in observing and controlling the dispersion of soil fungi, as a crucial measure to preserve public health is highlighted in this figure.

Opportunistic pathogenic fungi are frequently present in our immediate living environments, generating vast quantities of airborne spores. Consequently, susceptible populations, such as the elderly and immunocompromised patients, are exposed to various environmental fungal pathogens in the form of bioaerosols daily (Fig. 2). These populations have witnessed increased infection rates by groups of fungi, including *Aspergillus* spp. and Mucoromycotina species (in India), with resistance to antifungal treatments in the latter [74]. Molecular epidemiology studies have consistently shown that several fungal diseases are contracted from our surrounding environments [74,75]. This is particularly true for invasive fungal diseases (IFDs) caused by *Coccidioides* spp., *A. fumigatus*, and *Cryptococcus* spp. Fungal infections disproportionately impact specific regions worldwide, resulting in substantial morbidity and mortality (Fig. 1) [74].

Eumycetoma, a fungal form of mycetoma, is a high-priority disease that predominantly affects impoverished populations in sub-Saharan Africa. Treatment involves a 12-month regimen of costly broad-spectrum antifungals with only a 30% efficacy rate, frequently resulting in limb amputations. Diagnosing eumycetoma in low- and middle-income settings is challenging due to limited access to diagnostics, skilled personnel, and point-of-care tests, frequently resulting in under- or misdiagnosis. Utilizing broad-spectrum antifungals without confirmed diagnosis accelerates drug resistance, exacerbating the problem [76]. African nations face difficulties in diagnosing invasive aspergillosis, with *A. fumigatus* classified as a critical priority by the WHO.

Azole-resistant *A. fumigatus* rates, reaching 80–90% in Southeast Asia, are driven by the extensive use of azole fungicides in agriculture. Additionally, *Pneumocystis jirovecii*, a universally prevalent yeast-like fungus, causes a life-threatening form of pneumonia called Pneumocystis jirovecii pneumonia (PJP). This critical illness, associated with AIDS, is marked by a considerable global disease burden and high mortality rates, typically arising as CD4+ T cell counts decrease. PJP is most observed in HIV-negative individuals in conjunction with corticosteroid administration. Out of 52,364 specimens from 7504 patients submitted for microbiological evaluation, PJP was confirmed in 240 patients, with approximately 52% being HIV-positive. The overall in-hospital mortality rate was 25.4% and58% for ICU admissions [77].

The growing global health concern surrounding invasive fungal infections is exacerbated by the emergence of antifungal resistance and limited access to diagnostics and antifungal agents. The absence of surveillance data and specialized laboratories further aggravates these challenges. Antifungal resistance results in prolonged treatments, extended hospital stays, and a heightened demand for expensive, toxic second-line drugs. Such medications are frequently unattainable in low- and middle-income countries, leading to elevated mortality rates and underscoring the necessity for enhanced prevention and control initiatives [78].

#### 1.3.2 Antifungal resistance

The development of resistance is partially fuelled by improper antifungal application. The close connection between environmental populations of fungi and exposure to antifungals means that emerging environmental resistance is likely to impact the clinical management of fungal infections [79]. In the agricultural sector, plant pathogenic fungi persistently evolve resistance to various fungicides employed for their control. This rapid adaptation necessitates an ongoing cycle of innovation, as agribusinesses create modifications of existing fungicides or develop novel compounds to circumvent resistance build-up. Similarly, residues from antifungal veterinary medications may contaminate the environment, adversely affecting non-target fungi and crucial ecosystem functions. Notably, both licensed medical antifungals and agricultural/veterinary fungicides exhibit broad-spectrum activity across the fungal kingdom. Consequently, resistance emerges not only in crop pathogens but also in other environmental fungi, including potential human fungal pathogens [80].

**Fig. 3:**
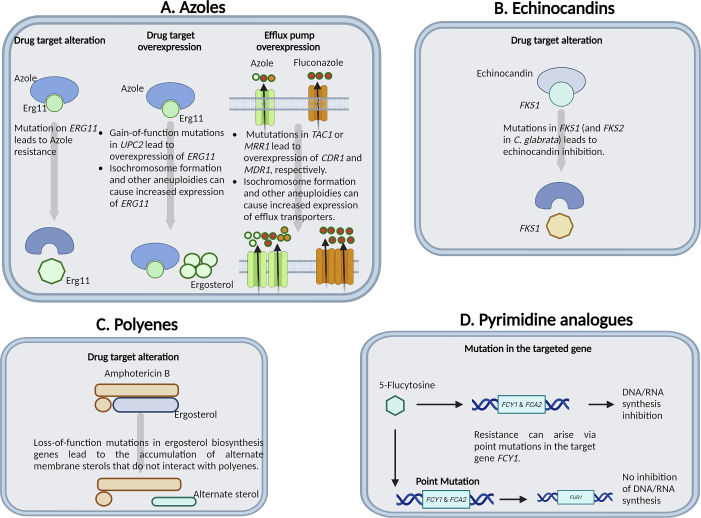
Pathways for developing antifungal drug resistance in fungal pathogens. **a |** Azole resistance in fungi is typically attributed to increased drug efflux from fungal cells, particularly in *Candida* species, and alterations to the sterol biosynthesis pathway resulting from point mutations and promoter insertions in the *CYP51A* gene, as observed in *Aspergillus fumigatus*. In species like *Cryptococcus neoformans*, chromosomal aneuploidy and hypermutation often drive drug target and efflux pump overexpression. Further resistance mechanisms involve alteration to the azole target Erg11, reducing the drug-binding affinity for the lanosterol demethylase enzyme. Resistance can be facilitated by overexpression of the drug target due to gain-of-function mutations in the *UPC2* transcriptional activator or through aneuploidies, specifically [i(5 L)], increasing *ERG11* copy number. Resistance is also established through upregulation of ABC transporters (green), including Cdr1 and Cdr2, activated by mutations in transcription factors like *TAC1* in *Candida* species and *PDR1* in *C. glabrata*. Finally, azole resistance can arise from overexpression of the MF transporter (brown), Mdr1, due to activating mutations in the *MRR1* transcription factor, or through efflux pump overexpression stimulated by aneuploidy formation. b Echinocandin resistance occurs due to changes in amino acids within key areas of the Fks subunits of glucan synthase, reducing the enzyme's sensitivity to the medication. **c |** Polyenes cause changes in cell membrane permeability by forming a complex with ergosterol, and resistance largely arises from loss-of-function mutations in ergosterol biosynthesis genes, especially within *Aspergillus* and *Candida* species. For *Candida albicans* specifically, *ERG3* dual loss triggers resistance, and drug tolerance is commonly associated with the upregulation of *ERG5, ERG6*, and *ERG25*. Ergosterol depletion primarily mediates polyene resistance, promoting alternate sterol production that does not interact effectively with polyenes, thus preventing extraction from the fungal cell membrane. **d |** Pyrimidine analogues such as 5-flucytosine hinder DNA and RNA synthesis. Resistance may develop through point mutations in the target gene *FCY1*, commonly seen in *Candida* species. Hypermutation in *Cryptococcus* species is also identified as a cause of resistance to this class of drugs template used from Fisher, M.C et all. 2022.

The One Health implications stemming from the pervasive use of agricultural fungicides, such as DMI azoles, have raised concerns regarding the potential emergence of resistance or tolerance in opportunistic fungi. These compounds bear structural similarity to medical triazoles and have experienced a surge in global usage (Fig. 3). In the United States, azole fungicide utilization increased by over 400% from 2006 to 2016, while China's usage was ten-fold higher, with comparable trends observed in the European Union. According to a WHO report, azole resistance rates in environmental samples reached 15–20% in certain European regions and exceeded 80% in Asia [74].

It is important to note that resistant strains can colonize healthy individuals, serving as potential reservoirs [81,82]. However, resistance rates exhibit considerable variation, contingent on animal species, geographical location, and methodologies employed for in vitro susceptibility testing.

### 1.4 Combating invasive fungal infections

The landscape of fungal pathogens causing human diseases continues to expand, leading to substantial morbidity and mortality. Simultaneously, the rise of resistance to antifungal drug families poses a growing challenge. Mechanisms underlying reduced susceptibility to azoles, such as those observed in *Candida albicans* and other *Candida* species, have been investigated extensively. Johnson and colleagues demonstrated synergistic effects of combining antifungal agents, highlighting the promise of enhanced treatment efficacy for fungal infections through strategic drug combinations.

These findings highlight the potential for similar approaches in human hosts, where understanding the role of fungal cell walls and capsules in immune response could lead to effective treatments and vaccines.

Further exploration of immunization strategies has revealed promising candidates. For instance, immunizing mice with the HIS-62 protein, isolated from *Histoplasma capsulatum* yeast cells, elicited a protective immune response, characterized by a significant population of monoclonal T-cells. This response was associated with the production of IL-10, IL-12, and IFN-γ. Recombinant protein Ag2/PRA and its truncations have displayed protective potential, while the secreted *Coccidioides*-specific antigen (CSA) has not yet demonstrated a protective response. Combining Ag2/PRA1–106 and CSA into a chimeric fusion protein has exhibited enhanced efficacy, increasing survival rates in mouse intranasal challenge models.

As efforts to counteract fungal infections intensify, a range of antifungal compounds are undergoing evaluation in clinical trials. In this dynamic landscape, novel antifungal agents are being developed to address the expanding array of fungal pathogens. Fosmanogepix, a prodrug of manogepix, holds promise with its potent, broad-spectrum activity against *Candida*, *Aspergillus*, and rare molds, including resistant strains. Ibrexafungerp, an oral glucan synthase inhibitor, presents fungicidal and fungistatic effects on *Candida* and *Aspergillus*, with potential against echinocandin-resistant *Candida*. Olorofim disrupts pyrimidine synthesis, exhibiting activity against specific fungi while minimizing toxicity. Opelconazole, an inhaled azole, targets pulmonary aspergillosis with potent activity against *Aspergillus* and *Candida* species.

**Fig. 4:**
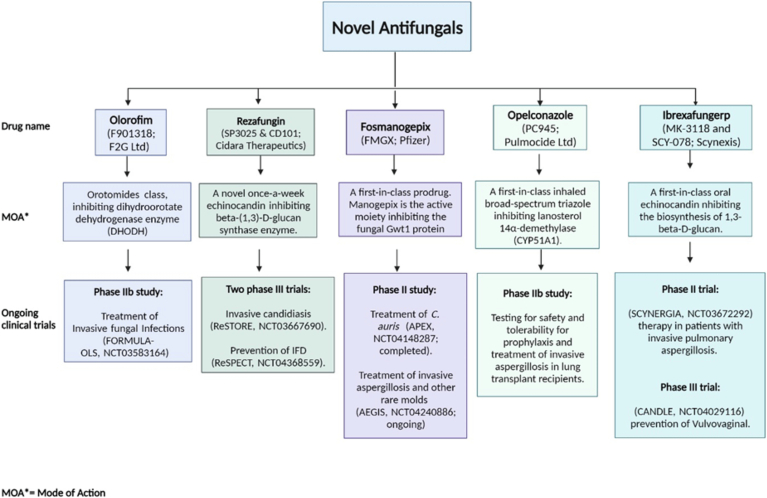
Overview of emerging antifungal agents. **Novel antifungal** drugs development landscape illustrating the diverse approaches being taken to address fungal infections.

In the face of evolving fungal threats and increasing drug resistance, research into novel antifungal agents and immunization strategies offers a multifaceted approach to tackle invasive fungal infections. By leveraging a combination of advanced therapies and a deeper understanding of host-fungus interactions, the medical community strives to enhance treatment outcomes and reduce the impact of these infections on public health (Fig. 4).

## 2 Conclusions

As a dominant group in soil, fungi strongly influence soil structure and, subsequently environment health. Limited information exists regarding the composition and diversity of the soil mycobiome. Recent culture-independent sequencing studies have broadened our knowledge around the diverse communities of the soil mycobiome. Fungal diversity tends to be higher in healthy soil than in disturbed or contaminated soil. Edaphic conditions, plant-microbe interactions, and biotic factors influence fungal population composition [83]. Agricultural practices have also been found to influence the composition of the soil mycobiome and affect plant health, suggesting the potential for managing and manipulating soil fungi to improve crop productivity [17]. Therefore, a deep understanding of the structure and function of the soil mycobiome is crucial to promote sustainable agricultural practices and ecosystem health, and to identify new targets for biological control of plant diseases and enhancing nutrient use efficiency in agroecosystems.

Despite the growing threat of fungal infections to human health, they continue to receive inadequate global attention and resources, complicating the accurate assessment of fungal infection prevalence, impeding policy and program development. While promising new antifungal drugs like Fosmanogepix, Olorofim are emerging, the simultaneous development of agricultural fungicides targeting the same pathway may heighten environmental resistance [84].

The available evidence presented herein stresses the importance of investigating the soil microbiome for gaining a deeper understanding of soil fungal ecology and its interplay with the rhizosphere microbiome, which carries significant implications for human health, animal health and environmental health.

Olorofim (F901318; F2G Ltd): Belonging to the orotomides class, this drug inhibits the dihydroorotate dehydrogenase enzyme. It is in a Phase IIb study for the treatment of invasive fungal infections (FORMULA-OLS, NCT03583164).

Rezafungin (SP3025 & CD101; Cidara Therapeutics): As a novel once-a-week echinocandin, it inhibits the beta-(1,3)-D-glucan synthase enzyme. It is involved in two Phase III trials for invasive candidiasis (ReSTORE, NCT03667690) and prevention of invasive fungal disease (IFD) (ReSPECT, NCT04368559).

Fosmanogepix (FMGX; Pfizer): This first-in-class prodrug's active moiety, manogepix, inhibits the fungal Gwt1 protein. It is under a Phase II study for the treatment of *C. auris* (APEX, NCT04148287; completed) and for invasive aspergillosis and other rare molds (AEGIS, NCT04240886; ongoing).

Opeleconazole (PC945; Pulmocide Ltd): A first-in-class inhaled broad-spectrum triazole that inhibits lanosterol 14α-demethylase (CYP51A1). It is in a Phase IIb study focused on safety and tolerability for prophylaxis and treatment of invasive aspergillosis in lung transplant recipients.

Ibrexafungerp (MK-3118 and SCY-078; Scynexis): This first-in-class oral echinocandin inhibits the biosynthesis of 1,3-beta-D-glucan. It is currently in a Phase II trial (SCYNERGIA, NCT03672292) for therapy in patients with invasive pulmonary aspergillosis and a Phase III trial (CANDLE, NCT04029116) for the prevention of vulvovaginal candidiasis.

**BOX 1: Timeline in the study of fungal diseases.**

Mycotic infections date back to the 18th century, with the first documented instance of *Aspergillus* infection in Paris in 1789. A link between oral candidiasis and *Candida albicans* was first established in 1839, and the inaugural account of mucormycosis was recorded in 1855. *Malassezia*, a causal agent of seborrheic dermatitis, was identified by Mallassez in 1874. In the late 19th century, several other significant mycological discoveries occurred. Coccidioidomycosis was first identified by a medical student in Argentina in 1892, followed by the discovery of blastomycosis in Baltimore, USA in 1894 and cryptococcosis in Germany that same year.

In the 20th century, the etiological agent of histoplasmosis was identified as a fungus, *Histoplasma* and *Pneumocystis* was subsequently implicated as a causative agent of pneumonia in humans. In a ground-breaking development in 1981, the first-ever reported pneumonia case in AIDS patients was ascribed to *Pneumocystis carinii*. The late 20th century was marked by notable mycotic outbreaks and the identification of new fungal pathogens. For instance, in 1994, dust from landslides induced by the Northridge earthquake resulted in an outbreak of coccidioidomycosis in Simi Valley, California. The same year, emergomycosis, a fatal systemic mycosis, was reported in an AIDS patient in Italy. Subsequent years witnessed a zoonotic epidemic of cat-associated sporotrichosis in Brazil (1998) caused by *S. brasiliensis* and a cryptococcosis outbreak due to *Cryptococcus gattii* on Vancouver Island, Canada (1999). In 2006, an outbreak of fungal keratitis caused by *Fusarium* species was associated with the use of a specific brand of contact lens solution across multiple states in the USA. In 2009, *C. auris*, was isolated in Japan from a patient with an ear infection. The early 21st century continued to unravel new fungal pathogens and mycotic outbreaks. In 2011, a necrotizing cutaneous mucormycosis outbreak was caused by the rare pathogen *Apophysomyces trapeziformis*, following a tornado in Missouri, USA. In 2012, *Saprochaete clavata*, an organism not previously recognized as a human pathogen, was responsible for fatal infections in multiple healthcare facilities in France. Simultaneously, an outbreak of fungal meningitis primarily caused by *Exserohilum rostratum*, a very rare human pathogen, was associated with patients who had received contaminated steroid injections. Finally, a notable development occurred in 2020, where, amidst the global COVID-19 pandemic, the first reports of fungal diseases associated with COVID-19 surfaced, including instances of COVID-19-associated pulmonary aspergillosis (CAPA) and an unprecedented surge in COVID-19-associated mucormycosis has further challenged the health care system in India in early 2021.

Alt-text: Unlabelled Box

**Figure:**
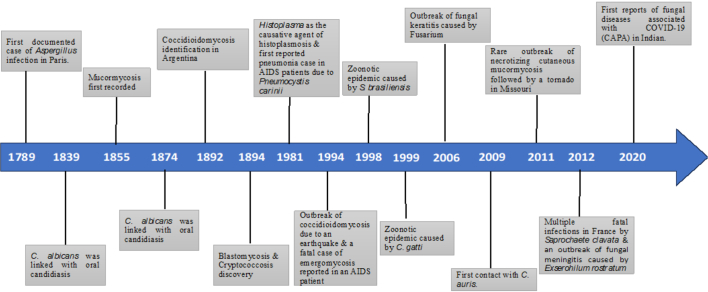

**Box 2: Combating invasive fungal infections.**

The landscape of fungal pathogens causing human diseases continues to expand, leading to substantial morbidity and mortality. Simultaneously, the rise of resistance to antifungal drug families poses a growing challenge. Mechanisms underlying reduced susceptibility to azoles, such as those observed in *Candida albicans* and other *Candida* species, have been investigated extensively. Johnson and colleagues demonstrated synergistic effects of combining antifungal agents, highlighting the promise of enhanced treatment efficacy for fungal infections through strategic drug combinations.

Critical interactions between fungi and hosts occur at the cell wall and capsule, which serve as initial contact points with host surfaces. Various fungal polysaccharides have been identified as pathogen-associated molecular patterns (PAMPs), recognized by pattern recognition receptors (PRRs). These receptors, transmembrane proteins, orchestrate signalling pathways, microbicidal activity, and phagocytosis, actively contributing to the host's innate immune response. *Blastomyces dermatitidis* utilizes BAD1, a surface adhesin, to evoke cell-mediated and humoral immune responses. While recombinant BAD1 immunization extends mouse survival, ultimate succumbing to infection remains common. Monoclonal antibodies, such as 2G8, have shown potential as immunotherapy against *B. dermatitidis* infections, improving survival rates in mice. The use of attenuated BAD1 null mutant vaccines effectively prevented fatal pulmonary infections and conferred acquired immunity in mice.

Further exploration of immunization strategies has revealed promising candidates. For instance, immunizing mice with the HIS-62 protein, isolated from *Histoplasma capsulatum* yeast cells, elicited a protective immune response, characterized by a significant population of monoclonal T-cells. This response was associated with the production of IL-10, IL-12, and IFN-γ. Recombinant protein Ag2/PRA and its truncations have displayed protective potential, while the secreted *Coccidioides*-specific antigen (CSA) has not yet demonstrated a protective response. Combining Ag2/PRA1–106 and CSA into a chimeric fusion protein has exhibited enhanced efficacy, increasing survival rates in mouse intranasal challenge models.

As efforts to counteract fungal infections intensify, a range of antifungal compounds are undergoing evaluation in clinical trials. In this dynamic landscape, novel antifungal agents are being developed to address the expanding array of fungal pathogens. Fosmanogepix, a prodrug of manogepix, holds promise with its potent, broad-spectrum activity against *Candida*, *Aspergillus*, and rare molds, including resistant strains. Ibrexafungerp, an oral glucan synthase inhibitor, presents fungicidal and fungistatic effects on *Candida* and *Aspergillus*, with potential against echinocandin-resistant *Candida*. Olorofim disrupts pyrimidine synthesis, exhibiting activity against specific fungi while minimizing toxicity. Opelconazole, an inhaled azole, targets pulmonary aspergillosis with potent activity against *Aspergillus* and *Candida* species.

In the face of evolving fungal threats and increasing drug resistance, research into novel antifungal agents and immunization strategies offers a multifaceted approach to tackle invasive fungal infections. By leveraging a combination of advanced therapies and a deeper understanding of host-fungus interactions, the medical community strives to enhance treatment outcomes and reduce the impact of these infections on public health.

Alt-text: Unlabelled Box

**Figure:**
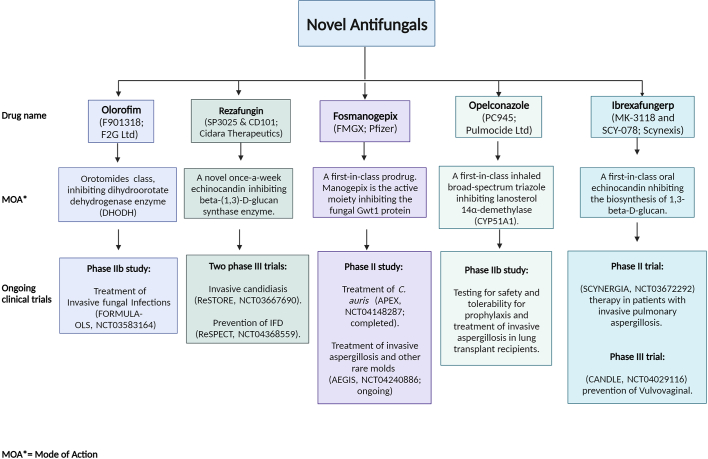

## Funding

None.

## Declaration of competing interest

All authors declare no conflict of interest.

## Data Availability

No data was used for the research described in the article.

## Appendix A Supplementary data

Supplementary material 1 Image 1

Supplementary material 2 Image 2
