# Supplementary material for: Fungal diversity in the soil Mycobiome: Implications for ONE health
Source: One Health. 2024 Apr 16;18:100720. doi: 10.1016/j.onehlt.2024.100720 (PMC11064618; doi:10.1016/j.onehlt.2024.100720)
Supplement: Supplementary file 1 — Supplementary material 1 [file mmc1.docx]

**Supplementary Figure 1: Timeline in the study of fungal diseases.**

Mycotic infections date back to the 18th century, with the first documented instance of *Aspergillus* infection in Paris in 1789. A link between oral candidiasis and *Candida albicans* was first established in 1839, and the inaugural account of mucormycosis was recorded in 1855. *Malassezia*, a causal agent of seborrheic dermatitis, was identified by Mallassez in 1874. In the late 19th century, several other significant mycological discoveries occurred. Coccidioidomycosis was first identified by a medical student in Argentina in 1892, followed by the discovery of blastomycosis in Baltimore, USA in 1894 and cryptococcosis in Germany that same year.

In the 20th century, the etiological agent of histoplasmosis was identified as a fungus, *Histoplasma* and *Pneumocystis* was subsequently implicated as a causative agent of pneumonia in humans. In a ground-breaking development in 1981, the first-ever reported pneumonia case in AIDS patients was ascribed to *Pneumocystis carinii*. The late 20th century was marked by notable mycotic outbreaks and the identification of new fungal pathogens. For instance, in 1994, dust from landslides induced by the Northridge earthquake resulted in an outbreak of coccidioidomycosis in Simi Valley, California. The same year, emergomycosis, a fatal systemic mycosis, was reported in an AIDS patient in Italy. Subsequent years witnessed a zoonotic epidemic of cat-associated sporotrichosis in Brazil (1998) caused by *S. brasiliensis* and a cryptococcosis outbreak due to *Cryptococcus gattii* on Vancouver Island, Canada (1999). In 2006, an outbreak of fungal keratitis caused by *Fusarium* species was associated with the use of a specific brand of contact lens solution across multiple states in the USA. In 2009, *C. auris*, was isolated in Japan from a patient with an ear infection. The early 21st century continued to unravel new fungal pathogens and mycotic outbreaks. In 2011, a necrotizing cutaneous mucormycosis outbreak was caused by the rare pathogen *Apophysomyces trapeziformis*, following a tornado in Missouri, USA. In 2012, *Saprochaete clavata*, an organism not previously recognized as a human pathogen, was responsible for fatal infections in multiple healthcare facilities in France. Simultaneously, an outbreak of fungal meningitis primarily caused by *Exserohilum rostratum*, a very rare human pathogen, was associated with patients who had received contaminated steroid injections. Finally, a notable development occurred in 2020, where, amidst the global COVID-19 pandemic, the first reports of fungal diseases associated with COVID-19 surfaced, including instances of COVID-19-associated pulmonary aspergillosis (CAPA) and an unprecedented surge in COVID-19-associated mucormycosis has further challenged the health care system in India in early 2021.
