# Supplementary material for: Fungal diversity in the soil Mycobiome: Implications for ONE health
Source: One Health. 2024 Apr 16;18:100720. doi: 10.1016/j.onehlt.2024.100720 (PMC11064618; doi:10.1016/j.onehlt.2024.100720)
Supplement: Supplementary file 2 — Supplementary material 2 [file mmc2.docx]

**Table 1: Distribution and occurrence of microbes in soil**

| **Fungi** | **Climate Zone** | **Vegetation Type** | **Soil Type** | **Ecological Role** | **Local Environmental, Conditions** | **Pathogenicity to humans** | **Transmission** | **Citation** |
| --- | --- | --- | --- | --- | --- | --- | --- | --- |
| *Aspergillus* spp. | Tropical | Rainforest | Loamy | Decomposer | High organic matter content | Aspergillosis, Aspergillomas | Inhalation | ^1^ |
| *Penicillium* spp. | Temperate | Grassland | Sandy | Decomposer | Moderate organic matter content |  |  | ^2^ |
| *Histoplasma capsulatum* | Various | Agricultural land | Sandy,  Loamy | Decomposer | Contaminated soils with birds and bats droppings, nitrogen & phosphorous rich soil | Histoplasmosis | Inhalation | ^3^ |
| *Exserohilum rostratum* | Tropical, Subtropical | Grassland | Various | Decomposer | High organic matter and moisture | Fungal Meningitis,  Fungal Sinusitis | Inhalation | ^4^ |
| *Rhizopus spp.* | Humid | Mixed vegetation | Clay | Mutualist | High moisture content | Mucormycosis | Inhalation,  Skin break | ^5^ |
| *Amanita muscaria* | Boreal | Coniferous forest | Acidic | Mycorrhizal symbiont | High acidity, low nutrient content | - | - | ^2^ |
| *Trichoderma* spp. | Subtropical | Agricultural land | Silty | Biocontrol agent | High nutrient content | Invasive pulmonary infection, CNS infection | Inhalation | ^6^ |
| *Morchella esculenta* | Temperate | Deciduous forest | Rich in humus | Mycorrhizal symbiont | High organic matter content | - | - | ^7^ |
| *Fusarium* spp. | Subtropical | Agricultural land | Sandy loam | Plant pathogen | Irrigated, crop rotation | Fusariosis | Inhalation,  Skin break |  |
| *Laccaria bicolor* | Temperate | Forest | Well-drained | Mycorrhizal symbiont | High organic matter content | - | - |  |
| *Phanerochaete chrysosporium* | Temperate | Deciduous forest | Loamy | Lignin decomposer | High lignin content, decaying wood | - | - |  |
| *Candida* spp. | Various | Various | Various | Opportunistic pathogen | Associated with human activity | Candidiasis,  Esophageal candidiasis,  Hepatosplenic candidiasis | Direct contact |  |
| *Cladosporium* spp. | Temperate | Forest | Loamy | Airborne, decomposer | High humidity, shaded areas | CNS infections,  Cutaneous infections | Inhalation |  |
| *Glomus intraradices* | Arid | Desert plants | Sandy | Arbuscular mycorrhizal fungi | Nutrient-poor, low moisture | - | - |  |
| *Beauveria* spp. | Various | Agricultural land | Various | Entomopathogenic fungi | Insect populations present | - | - |  |
| *Xylaria polymorpha* | Temperate | Deciduous forest | Organic-rich | Wood decomposer | Decaying wood, high humidity | - | - |  |
| *Tuber melanosporum* | Mediterranean | Oak and hazel | Calcareous | Mycorrhizal symbiont | High calcium content, well-drained | - | - |  |
| *Ophiocordyceps unilateralis* | Tropical | Rainforest | Organic-rich | Entomopathogenic fungi | High humidity, ant populations | - | - |  |
| *Purpureocillium lilacinum* | Various | Forests, Deserts, Grasslands | Sandy, Loamy, Clay | Nematode pathogenic fungus | High organic matter, High humidity, Nematode populations | Pulmonary, soft-tissue and disseminated infection in immunocompromised patients | Inhalation,  Direct contact |  |
| *Piriformospora indica* | Various | Agricultural land, Forests,Grassland | Sandy, Loamy, Clay | Symbiotic relationship with plant roots, Plant growth promoter | Low moisture, Arid | - | - | ^8^ |
| *Pochonia chlamydosporia* | Temperate | Agricultural Land, Forests | Sandy, Loamy | Nematode pathogenic fungus, Plant growth promoter | Nematode populations, Nutrient-poor, adverse soil conditions | - | - | ^2^ |
| *Mucor* spp. | Temperate | Various | Sandy, Loamy, Clay | Saprotrophs, Nutrient Cycling | High organic matter, High humidity | Mucormycosis | Inhalation,  Skin break | ^9^ |
| **Bacteria** |  |  |  |  |  |  |  |  |
| *Azospirillum brasilense* | Tropical | Grasses | Legumes, Sandy loamy | Nitrogen fixation | High organic matter | - |  | ^2^ |
| *Nitrosomonas europaea* | Temperate | Forests | Grasses, Loamy, Clayey | Nitrification | Well-aerated soils | - | - |  |
| *Pseudomonas putida* | All climates | Various | Various | Degradation of pollutants | Contaminated sites | Bacteraemia, fever in immunocompromised patients | Direct contact,  Catheter-related infection |  |
| *Arthrobacter globiformis* | All climates | Various | Loamy, Clayey, | Degradation of pesticides | Contaminated sites | - | - |  |
| *Frankia alni* | All climates | Alder trees | Loam, Sandy loam | Nitrogen fixation | Symbiotic association | - | - |  |
| *Blastocatella fastidiosa* | All climates | Savanna | Sandy loam | Undefined | Nitrogen-poor, semi-arid soils | - | - |  |
| *Sphingobacterium prati* | Temperate | Various | Meadow | Lignocellulose deconstruction | High organic matter content | - | - | ^10^ |
| *Rhizobium leguminosarum* | Temperate | Agriculture Land | Legumes, Loamy | Rhizobium-legume symbiosis | High organic matter, well-drained | - | - | ^2^ |
| *Burkholderia cepacia* | Mediterranean | Ryegrass | Grasses, Legumes | Plant growth promoter | Well-aerated soils | Respiratory infections in immunocompromised patients | Inhalation,  Direct contact,  Use of urinary & venous catheters |  |
| *Myxococcus xanthus* | Temperate | Various | Loamy, Sandy, Clay | Epibiotic predation of soil bacteria and fungi | High organic matter, Moderate moisture | - | - |  |
| **Protist** |  |  |  |  |  |  |  |  |
| *Acanthamoeba castellanii* | Temperate | Grasslands | Loam | Predation decomposition | High organic matter content | Acanthamoeba keratitis  Skin and lung infections | Direct contact  Skin break | ^11^ |
| *Paramecium bursaria* | Tropical | Rainforest | Clay | Mutualism predation | High humidity dense plant cover | - |  | ^12^ |
| *Cercomonas* spp. | Arid | Desert shrubs | Sandy | Predation decomposition | Low moisture extreme temperature | - | - | ^13^ |
| *Euglena longa* | Polar | Tundra | Peat | Photosynthesis grazing | Low temperature permafrost | - |  | ^14^ |
| *Vampyrella lateritia* | Temperate | Wetlands | Organic-rich soil | Predation decomposition | High nutrient levels wet conditions | - | - | ^15^ |
| *Gregarinomorphea* | Various | Forest, Vineyard | Various | Nutrient cycling | Well aerated topsoil, Neutral pH | - | - | ^16^ |
| *Polymyxa graminis* | Various | Agriculture Land | Loamy, Sandy | Plant root parasite | Well aerated, Adequate moisture | - |  | ^17^ |
| *Neoheteromita globosa* | Temperate | Grassland | Various | Nutrient cycling and bacterial parasites (bacterivorous) | High organic matter content | - | - | ^18^ |
| *Rhogostomidae* | Temperate | Agriculture Land | Loamy | Nutrient cycling and bacterial parasite | Adequate moisture,  High organic matter content | - | - | ^19^ |
| **Viruses** |  |  |  |  |  |  |  |  |
| Narnaviridae | Tropical | Rainforest | Clay | Fungal parasite | High humidity  dense plant cover | - | - | ^20^ |
| Partitiviridae | Arid | Desert shrubs | Sandy | Fungal and plant parasite | Low moisture extreme temperature | - | - | ^20^ |
| Inoviridae | Polar | Tundra | Peat | Bacterial parasite | Low temperature permafrost | - | - | ^21^ |
| Picobirnaviridae | Subtropical | Forest | Silty | Animal parasite | High rainfall, diverse flora fauna | Gastrointestinal infections | Zoonotic transmission,  Faecal-contaminated raw sewage | ^22^ |
| Phycodnaviridae | Temperate | Wetlands | Organic-rich soil | Algal parasite | High nutrient levels, wet conditions | - | - | ^23^ |
| Podoviridae | Polar | Tundra | Peat | Bacterial parasite | Arid Low temperature permafrost | Associated in the gut of patients with diarrhoea-predominant Irritable Bowel Syndrome (IBS) | Undefined | ^21^ |
| Mimiviridae | All climates | Forest | Silty | Protists and amoeba parasite | High organic matter | - | - | ^24^ |
| Reoviridae | All climates | Grassland | Prairie | Eukaryotic parasite (vertebrates & invertebrates) | High moisture | Respiratory infections  Gastroenteritis | Inhalation  Ingestion  Direct contact  Faecal-oral route | ^25^ |
| Siphoviridae | Polar | Tundra | Peat | Bacterial and archaeal parasite | Arid Low temperature permafrost | - | - | ^21^ |

**Table references**

1 Hättenschwiler S, Coq S, Barantal S, Handa IT. Leaf traits and decomposition in tropical rainforests: revisiting some commonly held views and towards a new hypothesis. *New Phytol* 2011; **189**: 950–65.

2 Beneficial Microbes in Agro-Ecology - 1st Edition. https://shop.elsevier.com/books/beneficial-microbes-in-agro-ecology/amaresan/978-0-12-823414-3 (accessed Sept 11, 2023).

3 Gómez LF, Torres IP, Jiménez-A M del P, *et al.* Detection of Histoplasma capsulatum in Organic Fertilizers by Hc100 Nested Polymerase Chain Reaction and Its Correlation with the Physicochemical and Microbiological Characteristics of the Samples. *Am J Trop Med Hyg* 2018; **98**: 1303–12.

4 Shoff CJ, Perfect JR. Uncommon Yeasts and Molds Causing Human Disease. In: Zaragoza Ó, Casadevall A, eds. Encyclopedia of Mycology. Oxford: Elsevier, 2021: 813–34.

5 Prakash H, Ghosh AK, Rudramurthy SM, *et al.* A prospective multicenter study on mucormycosis in India: Epidemiology, diagnosis, and treatment. *Medical Mycology* 2019; **57**: 395–402.

6 Harman GE, Howell CR, Viterbo A, Chet I, Lorito M. Trichoderma species — opportunistic, avirulent plant symbionts. *Nat Rev Microbiol* 2004; **2**: 43–56.

7 Amaresan N, Senthil Kumar M, Annapurna K, Kumar K, Sankaranarayanan A, editors. Index. In: Beneficial Microbes in Agro-Ecology. Academic Press, 2020: 861–912.

8 Gill SS, Gill R, Trivedi DK, *et al.* Piriformospora indica: Potential and Significance in Plant Stress Tolerance. *Frontiers in Microbiology* 2016; **7**. https://www.frontiersin.org/articles/10.3389/fmicb.2016.00332 (accessed Sept 11, 2023).

9 Ziaee A, Zia M, Bayat M, Hashemi J. Identification of Mucorales isolates from soil using morphological and molecular methods. *Curr Med Mycol* 2016; **2**: 13–9.

10 Besaury L, Floret J, Rémond C. Sphingobacterium prati spp. nov., isolated from agricultural soil and involved in lignocellulose deconstruction. *Int J Syst Evol Microbiol* 2021; **71**: 004963.

11 Xuan Y, Shen Y, Ge Y, Yan G, Zheng S. Isolation and identification of Acanthamoeba strains from soil and tap water in Yanji, China. *Environ Health Prev Med* 2017; **22**: 58.

12 Spanner C, Darienko T, Filker S, Sonntag B, Pröschold T. Morphological diversity and molecular phylogeny of five Paramecium bursaria (Alveolata, Ciliophora, Oligohymenophorea) syngens and the identification of their green algal endosymbionts. *Sci Rep* 2022; **12**: 18089.

13 Bass D, Howe AT, Mylnikov AP, *et al.* Phylogeny and Classification of Cercomonadida (Protozoa, Cercozoa): Cercomonas, Eocercomonas, Paracercomonas, and Cavernomonas gen. nov. *Protist* 2009; **160**: 483–521.

14 Záhonová K, Füssy Z, Birčák E, *et al.* Peculiar features of the plastids of the colourless alga Euglena longa and photosynthetic euglenophytes unveiled by transcriptome analyses. *Sci Rep* 2018; **8**: 17012.

15 Anderson TR. Soil populations of the root rot fungal pathogen Chalara elegans and the mycophagous amoeba Vampyrella lateritia following soil fumigation. *Soil Biology and Biochemistry* 1993; **25**: 223–6.

16 Xue P, Minasny B, McBratney A, Jiang Y, Luo Y. Land use effects on soil protists and their top-down regulation on bacteria and fungi in soil profiles. *Applied Soil Ecology* 2023; **185**: 104799.

17 Tyagi S, Sultana R, Ju H-J, *et al.* The Development of Simple Methods for the Maintenance and Quantification of Polymyxa graminis. *Indian J Microbiol* 2016; **56**: 482–90.

18 Khanipour Roshan S, Dumack K, Bonkowski M, Leinweber P, Karsten U, Glaser K. Taxonomic and Functional Diversity of Heterotrophic Protists (Cercozoa and Endomyxa) from Biological Soil Crusts. *Microorganisms* 2021; **9**: 205.

19 Dumack K, Flues S, Hermanns K, Bonkowski M. Rhogostomidae (Cercozoa) from soils, roots and plant leaves (Arabidopsis thaliana): Description of Rhogostoma epiphylla spp. nov. and R. cylindrica spp. nov. *Eur J Protistol* 2017; **60**: 76–86.

20 Roossinck MJ. Evolutionary and ecological links between plant and fungal viruses. *New Phytologist* 2019; **221**: 86–92.

21 Zablocki O, van Zyl L, Adriaenssens EM, *et al.* High-Level Diversity of Tailed Phages, Eukaryote-Associated Viruses, and Virophage-Like Elements in the Metaviromes of Antarctic Soils. *Applied and Environmental Microbiology* 2014; **80**: 6888–97.

22 Kashnikov AYu, Epifanova NV, Novikova NA. On the nature of picobirnaviruses. *Vavilovskii Zhurnal Genet Selektsii* 2023; **27**: 264–75.

23 Alarcón-Schumacher T, Guajardo-Leiva S, Antón J, Díez B. Elucidating Viral Communities During a Phytoplankton Bloom on the West Antarctic Peninsula. *Front Microbiol* 2019; **10**: 1014.

24 Schulz F, Alteio L, Goudeau D, *et al.* Hidden diversity of soil giant viruses. *Nat Commun* 2018; **9**: 4881.

25 Wu R, Davison MR, Gao Y, *et al.* Moisture modulates soil reservoirs of active DNA and RNA viruses. *Commun Biol* 2021; **4**: 1–11.
